# Detection of maladaptive pain in dogs referred for behavioral complaints: challenges and opportunities

**DOI:** 10.3389/fnbeh.2025.1569351

**Published:** 2025-05-08

**Authors:** Jenthe Kwik, Tiny De Keuster, Tim Bosmans, José Mottet

**Affiliations:** ^1^Veterinary Behavioral Referral Practice – DAP Vigor, Essen, Belgium; ^2^Veterinary Specialist Behavioral Referrals, Lievegem, Belgium; ^3^Small Animal Department, Faculty of Veterinary Medicine, Ghent University, Merelbeke, Belgium; ^4^AUW REVOIR – Center for Pain Expertise, Oosterzele, Belgium

**Keywords:** dog behavior, behavioral signs, maladaptive pain, home video, time line, differential diagnosis, physical signs, red flags

## Abstract

**Introduction:**

Diagnosing maladaptive pain in dogs with behavioral complaints is challenging, as clinical signs are often non-specific and may be absent during examination. This paper supports veterinary teams in distinguishing behavioral changes that stem from a behavioral disorder, maladaptive pain, or both.

**Methods:**

The medical records of ten client-owned dogs referred to the authors’ behavioral practice were selected to identify challenges in recognizing maladaptive pain and to highlight diagnostic tools. A Toolbox approach was used for assessment, integrating caregiver questionnaires, medical history, home video analysis, clinical observation, and a timeline. Behavioral signs were categorized as green (adaptive) or red (maladaptive) flags to facilitate differential diagnosis and guide treatment decisions.

**Results:**

All dogs (*n* = 10) were diagnosed with altered socioemotional functioning. In 7/10 cases, the Toolbox approach indicated maladaptive pain, confirmed by medical imaging in 3/7 cases. Multimodal treatment led to recovery in 6/7 dogs. In 3/10 dogs with behavioral histories, an acute worsening of signs suggested maladaptive pain, confirmed by imaging in all cases. Treatment led to partial recovery in 2/3 dogs, while one was euthanized due to neoplasia. Diagnostic challenges fell into three categories: bias in observation, clinical examination, and interpretation of behavioral signs.

**Conclusion:**

Diagnosing maladaptive pain in dogs with behavioral problems requires a comprehensive approach. Recognizing red flags, using targeted diagnostic tools, and implementing multimodal treatment strategies can improve quality of life, reduce suffering, and enhance case management.

## Introduction

Pain management is a fundamental aspect of veterinary medicine, essential for ensuring well-being and quality of life of companion animals (Mathews et al., 2014; Monteiro et al., 2023). Despite its importance, the accurate assessment and diagnosis of pain remain challenging, particularly in cases of maladaptive pain (Belshaw and Yeates, 2018). Unlike acute or adaptive pain, which serves as a protective response to a noxious stimulus, maladaptive pain involves chronic alterations in pain processing, significantly affecting socioemotional functioning and the animal’s overall behavior (Greene, 2010; Cimino Brown, 2017).

Lascelles et al. (2019) highlighted that maladaptive pain affects multiple dimensions, including gait, movement, somatosensory processing, sleep, cognitive function, affective function, as well as yet-to-be-discovered dimensions. The multidimensional impact of maladaptive pain makes its assessment particularly challenging, as individual dogs respond differently to both pain and analgesics, further complicating assessment, and treatment. For instance, an analgesic might effectively address the sensory discomfort of pain but might fail to improve the affective or cognitive consequences of chronic pain, due to altered nociceptive processing (Lascelles et al., 2019).

Research over the past decades has identified the challenges clinicians face in diagnosing maladaptive or chronic pain in dogs (Wiseman-Orr et al., 2004; Belshaw and Yeates, 2018; Reid et al., 2018; Lascelles et al., 2019; Malkani et al., 2024). Maladaptive pain can be easily overlooked during clinical observation and examination, as most signs are non-specific and non-observable in a clinical setting. Therefore, information retrieved from caregivers is indispensable for diagnosis because caregivers can detect subtle changes in their dog’s day-to-day functioning in the home environment (Wiseman-Orr et al., 2004; Reid et al., 2018).

Another challenge lies in detecting the relationship between chronic pain and behavioral problems, as well as tracking the evolution of clinical signs over time. Mills et al. (2020) found that 30–80% of patients referred for behavioral complaints had at least one underlying painful condition, emphasizing the importance of screening behavioral patients for pain (Mills et al., 2020). Research by Malkani et al. (2024) noted that in dogs with musculoskeletal disorders, behavioral signs typically precede physical signs. In a study evaluating dogs over time using the Animal Welfare Assessment Grid (AWAG), behavioral signs, such as increased fearfulness, prolonged recovery after a stressful event, or reduced caregiver interaction, preceded physical signs, such as limping, lameness, or stiff gait (Malkani et al., 2024). Caregivers in this study were often unaware of these behavioral changes and delayed seeking veterinary care until physical signs became apparent (Malkani et al., 2024). The above-mentioned studies highlight the importance of diagnosing maladaptive pain to improve a dog’s well-being while revealing the challenges faced by veterinary teams during diagnosis.

This retrospective study aimed to explore the challenges in diagnosing maladaptive pain in dogs presenting with behavioral complaints. Additionally, it investigates the feasibility of a structured behavioral clinical reasoning protocol to distinguish the underlying cause of behavioral problems in dogs, whether this is a behavioral disorder, maladaptive pain, or a combination of both (Camps et al., 2019; Mills et al., 2020, 2023).

## Materials and methods

The medical records of 10 client-owned dogs, referred by general practice veterinarians (GPs) to a behavioral specialist and resident for behavioral examination, were selected to identify common challenges in recognizing maladaptive pain in these dogs and to highlight tools that can help address these challenges. The reasons for referral (behavioral complaints) were as follows: Dog 1 was presented for biting a family member, Dog 2, 4, and 5 for vocalizing at night and sleeping problems, Dog 3 for lunging toward unfamiliar dogs on walks, Dog 6 for biting the family dog, Dog 7 for pica, Dog 8 for biting family members, Dog 9 for panic attacks when hearing noises, and sleeping problems, and Dog 10 for snapping toward the caregiver on walks. The specific case details at the time of referral admission are outlined in Table 1.

**Table 1:** This table describes 10 client-owned dogs referred to a behavioral specialist or resident.

Dog	Dog characteristics	Medical history pre-behavioral consultation	Behavioral complaint = reason for referral
1	New Foundland, M, 7y	Medical history: recurrent lick granuloma right tarsus GP clinical exam: would growl, snap Direct observation: lies down, refuses to walk X-rays tarsus: NA Dermatological examination: NA Surgery: Biopsy and resection (2x)	*Biting a family member*
2	Sheltie, M, 8y	Medical history: NA GP clinical exam: NA Direct observation: NA X-rays: NA	Vocalizing at night and sleeping problems
3	Golden Retriever, FS, 3y	Medical history: right hindlimb limping GP clinical exam: painful on right hindlimb X-rays: hip dysplasia right hindlimb Surgery: right hind limb, femoral head resection Direct observation: post-surgery: NA X-rays post-surgery: NA	*Lunging toward unfamiliar dogs on walks*
4	Labrador Retriever Mn, 8y	Medical history: intermittent limping GP clinical exam: would growl, snap Direct observation: signs expected of hip dysplasia X-rays: bilateral hip dysplasia	*Vocalizing at night and sleeping problems*
5	Bulgarian Scenthound, M, 7y	Medical history: nodule on right hind leg GP clinical exam: painful when touched Direct observation: NA Neurological exam: NA, biopsy recommended Biopsy: Surgery: Amputation right hindleg because of malignant peripheral nerve sheath tumor (MNST) GP clinical exam: Healed as expected, NA	*Vocalizing at night and sleeping problems*
6	Corgi, M, 2y	Medical history: limping on hindlimbs GP clinical exam: painful hindlimbs Orthopedic exam: limping on hindlimbs X-rays: bilateral hip dysplasia Surgery: right hind limb, femoral head resection Surgery: left hind limb, femoral head resection X-rays post-surgery: NA Direct observation post-surgery: limping was seen as an expected sign in the process of revalidation	*Biting the family dog*
7	Labrador mix, M, 9 months	Medical history: intermittent limping GP Clinical exam: painful front & hindlimbs Direct observation: limping on front- and hindlimbs Orthopedic exam: painful elbows and hips X-rays: bilateral elbow dysplasia, bilateral severe hip dysplasia Surgery: 5x enterectomy following foreign body ingestion	*Pica*
8	Border Collie, M, 3y	Medical history: intermittent limping on left hindlimb GP clinical exam: would growl, snap Direct observation: NA	*Biting family members*
9	Malinois, Mn, 8y	Medical history: NA GP clinical exam: NA Direct observation: NA Orthopedic exam: NA X-rays hip region, back region: NA	*Panic attacks when hearing noises, and sleeping problems*
10	Husky, Mn, 8y	Medical history: intermittent limping on left front limb GP clinical exam: NA Direct observation: NA Orthopedic exam: NA X-rays front legs, hind legs, hips: NA Physiotherapist examination: suspected pain in left front limb	*Snapping toward caregiver on walks.*

The columns indicate, respectively, column 1, dog number; column 2, dog characteristics; and column 3, medical history pre-behavioral consultation with results of medical examinations, direct observation, medical imaging, or surgery. Column 4: Reasons for referral to a veterinary behavioral specialist or resident. GP, General practitioner; NA, No abnormalities found.

The functional approach to the behavioral examination used is referred to as the Toolbox approach and consists of 1/ background information including: 1a/ caregiver-completed questionnaires with open ended questions relating to the behavioral history and 1b/ the medical history with the information obtained from the clinical examination of the GP 2/ observation through home videos, 3/ chronological mapping of clinical signs (behavioral and physical) on a timeline 4/ caregiver interview 5/ differential diagnosis, 6/ diagnosis, 7/ treatment options, 8/follow up (De Keuster, 2022, 2025, unpublished).

Before mapping the clinical signs chronologically, they were assessed and categorized as green or red flags to detect potential social, emotional, physical, or cognitive (mal)functioning or disease processes (De Keuster, 2025, unpublished). Green flags are defined as observable behavioral signs that align with the dog’s characteristics and life stage, which are within a dog’s spectrum of typical functioning. Examples include eating, drinking, self-hygiene, resting, sleeping, elimination, play, and responses to familiar and unfamiliar social and non-social stimuli (humans, conspecifics, vehicles, noises, etc.). Green flags reflect behaviors that are flexible and adaptive in terms of frequency, duration, latency, and magnitude of response, and lead to recovery within a given context (De Keuster, 2025, unpublished). Red flags are defined as observable behavioral signs with respect to the dog’s characteristics and life stage, which are outside of a dog’s spectrum of typical functioning. Examples include maladaptive, inflexible, restrictive, and repetitive behaviors that are performed without recovery across different contexts. Red flags warrant further investigation to assess their underlying cause (De Keuster, 2025, unpublished).

## Results

The assessment of clinical signs for green and red flags resulted in the identification of changes in behavior patterns, such as eating, drinking, resting, sleeping, self-grooming, elimination, locomotion, solo-play, social play, caregiver-interaction, interaction with the family dog, behavior on walks, and responses to noises that might relate to the presence of maladaptive pain (red flags). These results are displayed in Table 2.

**Table 2:** Investigation of red (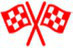) and green (V) flags in day-to-day functioning of the dog sample (*n* = 10).

Dog behaviors and contexts screened in behavioral examination	Dog 1	Dog 2	Dog 3	Dog 4	Dog 5	Dog 6	Dog 7	Dog 8	Dog 9	Dog 10
Eating/drinking	V	V	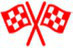	V	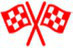	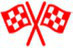	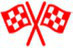	V	V	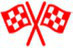
Resting	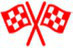	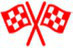	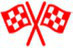	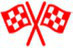	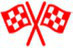	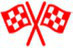	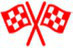	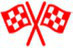	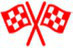	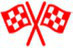
Sleeping	V	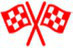	V	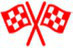	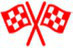	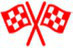	V	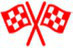	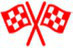	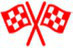
Self-grooming	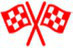	V	V	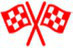	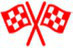	V	V	V	V	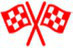
Elimination	V	V	V	V	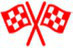	V	V	V	V	V
Locomotion	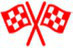	V	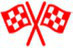	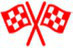	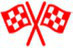	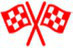	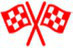	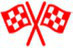	V	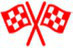
Solo play—object	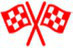	V	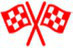	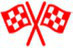	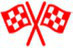	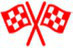	V	V	V	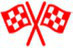
Social play—caregivers	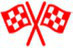	V	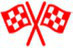	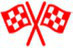	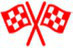	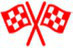	V	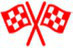	V	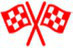
Interaction with caregivers	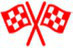	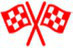	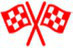	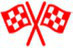	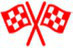	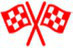	V	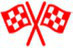	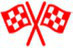	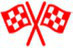
Interaction familiar dog	NA	NA	NA	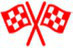	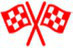	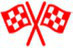	V	NA	NA	NA
Walks—meeting people	V	V	V	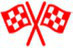	V	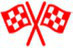	V	V	V	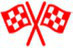
Walks—meeting dogs	V	V	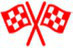	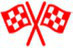	V	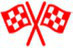	V	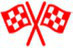	V	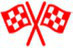
Walks—moving vehicles	V	V	V	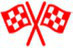	V	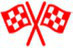	V	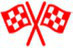	V	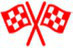
Response to noises	V	V	V	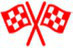	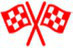	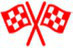	V	V	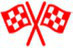	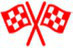

Clinical signs (green flags/red flags) were assessed and screened using caregiver questionnaires with open-ended questions reflecting the caregivers’ descriptions of the dog’s behavioral signs in the home setting and on walks. This information was cross-checked with home videos and face-to-face interviews with the caregivers. NA, Not Applicable.

Additionally, by using the Toolbox approach, several challenges were identified in diagnosing maladaptive pain in dogs presenting with behavioral signs, which were classified into three categories: biases in observation, biases in clinical examination, and biases in the interpretation of clinical signs. These challenges are displayed in Table 3.

**Table 3:** This table summarizes the challenges in diagnosing maladaptive pain in clinical settings of the 10 dogs described in Table 1, organized into three bias categories: observation, clinical examination, and interpretation of clinical signs.

	CHALLENGES in a clinical setting of dogs presented with behavioral complaints		
Dog	Bias in direct observation	Bias in clinical examination	Bias in interpretation of clinical signs
Dog 1	Dog lies down immediately, refuses to walk during consultation	Difficult to examine, would growl, snap	Licking, biting, can be highly suspicious of behavioral problems (in absence of medical diagnosis)
Dog 2	Walks normal in veterinary clinic would not show any sign of limping	Reluctant to being handled, tense body, shows no signs at the veterinary clinic	Sleeping problems were misinterpreted as being the caregiver’s perception (e.g., spoiled dog)
Dog 3	Walks normal in veterinary clinic would not show any sign of limping	Reluctant to being handled, tense body, shows no signs at the veterinary clinic	Pica, lunging at dogs, decreased play, decreased interaction with caregivers were misinterpreted as behavioral signs.
Dog 4	Known history of hip dysplasia, limping was seen as expected sign in this dog	Reluctant to being handled at the veterinary clinic, lies down easily, would growl when being touched	Insomnia, restlessness, vocalizing and altered interaction with family dog and caregiver were misinterpreted as behavioral signs
Dog 5	Increased licking at healed surgical site was seen as normal postoperative behavior No signs of skin damage or pain were observed	Clinical exam post-amputation revealed no abnormalities	Vocalizing, social isolation, irritability, and urination were misinterpreted as behavioral signs
Dog 6	Limping was observed post-surgery, but this was seen as an expected sign in this dog in the process of revalidation	Clinical and radiological exam post-surgery revealed no abnormalities	Increased irritability toward the family dog, decreased interaction with caregivers, and apathy were misinterpreted as behavioral signs
Dog 7	No significant challenges during observation	No significant challenges during the clinical exam, osteoarthritis was diagnosed by radiographs	Pica was interpreted as a purely behavioral sign
Dog 8	Normal gait observed, would not show signs of limping during consultation	Difficult to examine without sedation, would growl, snap, and bite, could not be muzzled. Range of motion of hip joints was normal under sedation	Biting the caregivers was misinterpreted as a purely behavioral sign after no improvement in behavioral signs was seen after a 10-day NSAID trial. The dog also had an active lifestyle and was young (3y) which, for the GP vet, indicated that there was no pain.
Dog 9	Normal gait observed during consultation	No significant challenges during the clinical examination	Increasing noise sensitivity, sleeplessness, panting, and panic reactions were seen as behavioral signs
Dog 10	Tense body posture and normal gait observed during consultation	Reluctant to being handled, a tense body, would growl, snap, and bite. Exam with muzzle revealed no abnormalities	The increased separation related problems, restlessness, and impaired social behavior were seen as purely behavioral signs because of the behavioral history of the dog.

The cases were split into two groups: group 1 (Dog 1–7): dogs without a behavioral history, and group 2 (Dog 8–10): dogs with a behavioral history and acute worsening of signs.

Differential diagnoses and recommendations for further examinations were based on the interpretation of signs after using the Toolbox approach and gathering all available information from questionnaires, home videos, and interviews, which were mapped onto a timeline. In Group 1 (dogs 1–7), red flags could not be explained by behavioral history, indicating a high likelihood of unidentified maladaptive pain. Group 2 (dogs 8–10) showed a behavioral history, but the Toolbox approach revealed an unexplained deterioration in behavioral signs, suggesting an underlying maladaptive pain process. Details of the diagnosis, investigation, treatment, and follow-up for Groups 1 and 2 are provided in Tables 4, 5, respectively.

**Table 4:** Behavioral red flags in dogs 1–7 could not be explained by a behavioral history; neither could impaired social functioning be explained by an underlying emotional disorder or cognitive dysfunction.

	Diagnosis behavioral examination	Type of referral investigation & results	Treatment approach and follow up
Dog 1	No indications of underlying emotional or cognitive disorder. Clinical signs highly indicative of unidentified maladaptive pain	*Medical imaging: CT scan* Osteochondrosis dissecans of the seventh lumbar vertebra (L7) with compression of the cauda equina Right sided foraminal stenosis of L7	Physical comfort: orthopedic bed Maladaptive pain: gabapentin 20 mg/kg TID Impaired socioemotional functioning/compulsive licking: SSRI fluoxetine 1.5 mg/kg SID Follow Up + 6 months: reduction red flags Follow up 3 yrs.: acute lymphoma euthanized at age 10 yrs.
Dog 2	No indications of underlying emotional / cognitive disorder. Clinical signs highly indicative of unidentified maladaptive pain	*Medical imaging: CT scan* Cervical: disc bulging C3-C4 with compression of the ventral spinal cord Hips: degenerative joint disease of the left hip Lumbar region: disc bulging T12-T13 with ventral compression of the spinal cord	Physical comfort: orthopedic bed Maladaptive pain: gabapentin 20 mg/kg TID Impaired socioemotional functioning (sleeping pattern) SSRI sertraline 2 mg/kg BID Follow up + 6 years: recovery, absence red flags still on medication; now age 17 yrs.
Dog 3	No indications of underlying emotional / cognitive disorder. Clinical signs highly indicative of maladaptive pain	*No further examinations conducted* Based on behavioral examination: indicative of maladaptive, postoperative pain	Physical comfort: orthopedic bed, physiotherapy, swimming 1x/week Maladaptive pain: pregabalin 2 mg/kg BID Impaired socioemotional functioning SSRI fluoxetine 1.5 mg/kg SID Follow up 6 months: complete recovery, absence of red flags Follow up +4 years: acute PRA (Progressive Retinal Atrophy) euthanized at age 7 years
Dog 4	No indications of underlying emotional / cognitive disorder. Clinical signs highly indicative of unidentified maladaptive pain	*Medical imaging: radiographs* Bilateral hip dysplasia Suspicion of lumbosacral stenosis	Physical comfort: orthopedic bed Maladaptive pain: gabapentin 20 mg/kg TID Impaired socioemotional functioning SSRI fluoxetine 1.5 mg/kg SID Follow up 6 months: reduction red flags, good sleep quality Follow up +4 years: euthanized at age 12 years because of other, unrelated medical problems
Dog 5	No indications of underlying emotional / cognitive disorder. Clinical signs highly indicative of unidentified maladaptive pain	*No further examinations conducted* Diagnosis: neuropathic / maladaptive pain post-surgery was suspected. Correlation between pain, pica, decreased play behavior and decreased interaction with caregivers was suspected.	Physical comfort: orthopedic bed Maladaptive pain: gabapentin 20 mg/kg TID Impaired socioemotional functioning SSRI fluoxetine 1.5 mg/kg SID Follow up 6 months: reduction all red flags, good sleep quality, no more complaints Follow up 2 years: euthanized at age 9 years because of recurrence MNST
Dog 6	No indications of underlying emotional / cognitive disorder. Clinical signs highly indicative of unidentified maladaptive pain	*No further examinations conducted* Diagnosis: neuropathic / maladaptive pain post-surgery was suspected. Correlation with impaired social functioning toward family dog was evidenced in the Timeline.	Management: 100% separation from other dog Physical comfort: orthopedic bed Maladaptive pain: gabapentin 20 mg/kg TID Impaired socioemotional functioning SSRI fluoxetine 1.5 mg/kg SID No improvement, caregiver stopped treatment
Dog 7	No indications of underlying emotional / cognitive disorder. Clinical signs highly indicative of unidentified maladaptive pain	*No further examinations conducted* Diagnosis: severe hip dysplasia and elbow dysplasia was documented. Correlation between maladaptive pain and pica was suspected.	Physical comfort: orthopedic bed Maladaptive pain: gabapentin 20 mg/kg TID Follow up 6 months: no more signs of pica Follow up +2 yrs.: dog still on gabapentin

Red flags in dogs 1–7 were found to be highly indicative of unidentified maladaptive pain, and dogs 1–7 were referred for further screening of maladaptive pain.

**Table 5:** Dogs 8–10 all had a behavioral history, respectively, dog 8: problems with conspecifics since age 1 year and in the timeline acute increase of red flags in responses toward vehicles and family members; dog 9: problems for years when exposed to loud noises, or thunderstorms, and these had abruptly evolved toward panic attacks (climbing on furniture, escape attempts) and recent sleeping problems (panic attacks even when all is quiet); dog 10: had problems for years when being separated from caregiver, and in the timeline abrupt increase of red flags over the past 4 months the dog showed red flags in all behaviors screened.

	Results behavioral examination	Type of referral investigation & results	Treatment approach and follow up
Dog 8	History of impaired socioemotional functioning with *acute worsening* of clinical signs highly indicative of maladaptive pain.	*Medical imaging: radiographs:* Bilateral hip dysplasia with remodeling of the femoral heads and necks, particularly significant in the left hip	Safety management: caregiver awareness, allowing dog space on its own, predictable interactions only Physical comfort: orthopedic bed Maladaptive pain: gabapentin (20 mg/kg TID) Anti-inflammatory pain management: robenacoxib (4 mg/kg SID) Significant improvement but does still growl when touched unexpectedly
Dog 9	History of impaired socioemotional functioning with *acute worsening* of clinical signs highly indicative of maladaptive pain.	*Medical imaging: CT scan* High grade degenerative lesions, calcifications and disc bulging with spinal cord compression at multiple levels including cervical, thoracic, and lumbar regions Impingement at the attachment of the biceps tendon in the right shoulder	Panick attacks and maladaptive pain: several drugs were tried to reduce signs of panic attacks, resp.: SSRI (fluoxetine 2 mg/kg SID + gabapentin 20 mg/kg TID (no response), addition of SARI (trazodone): no improvement TCA (amitriptyline) + pregabalin (4 mg/kg/BID): fair results at nighttime (relaxed sleep), but dog still shows trembling and restlessness when hearing loud noises
Dog 10	History of impaired socioemotional functioning with *acute worsening* of clinical signs highly indicative of maladaptive pain.	*Medical imaging: radiographs:* No abnormalities *Medical imaging: CT scan* Revealed lesions with high suspicion of malignant peripheral nerve sheet tumor of left front leg	Options for treatment were discussed, from surgery to medication (tramadol etc.). Because of reduced welfare and red flags in all sections, due to escalating pain, the caregiver decided on euthanasia.

To illustrate clinical reasoning and the importance of using a timeline for the interpretation of clinical signs (red and green flags), the cases are described in more detail.

### Dog 1: New Foundland dog, M, 7y

**Figure 1:**
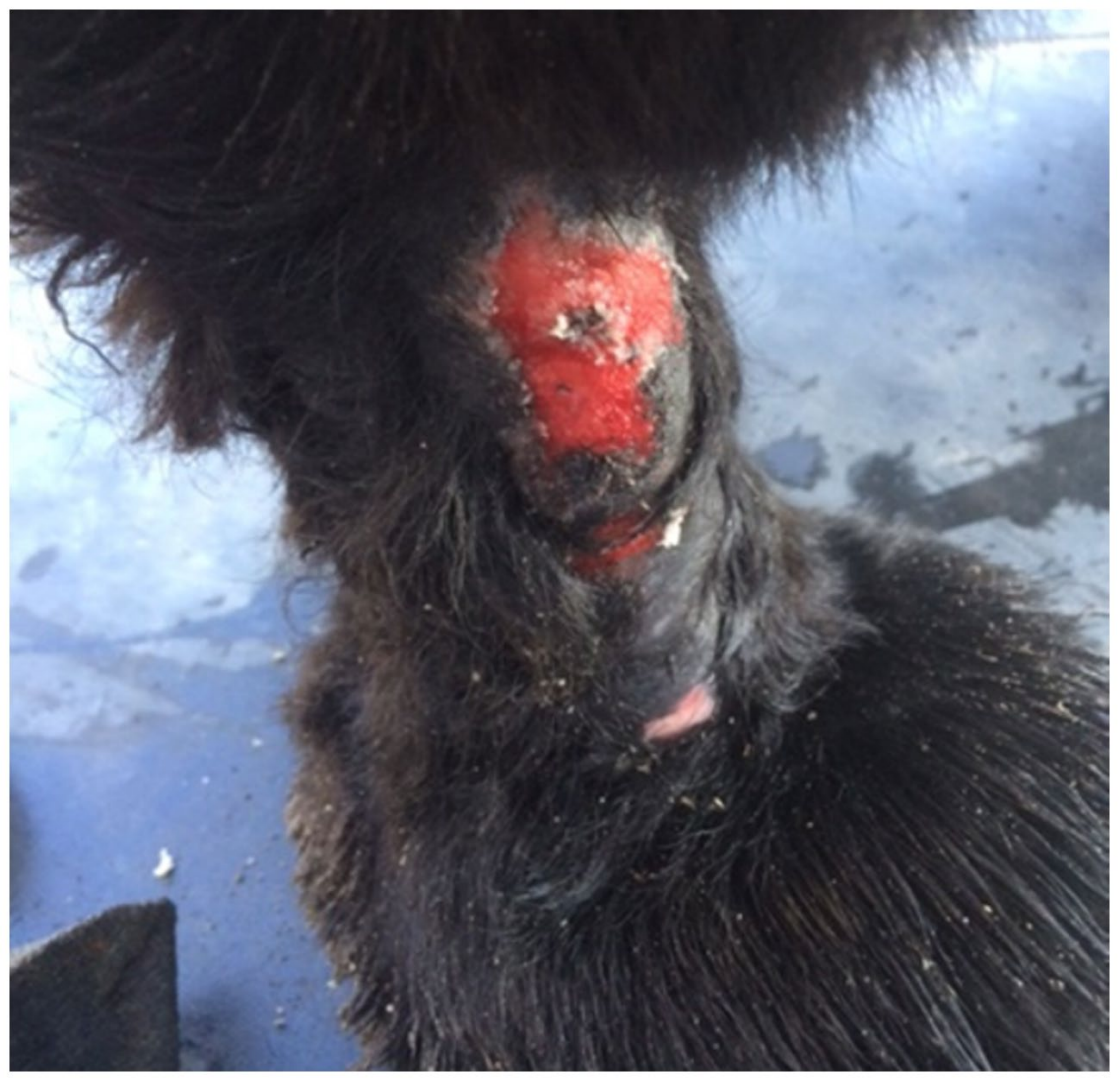
Dog 1: Right tarsal lick granuloma resulting from excessive licking behavior.

Dog 1 had a recurrent lick granuloma on the right tarsus for 3 years (Figure 1), with dermatological, biopsy, and radiographic results showing no abnormalities. Surgical resections and radiographs revealed no improvement or underlying pathology. During direct observation, the dog laid down and refused to walk. The GP clinical exam noted discomfort, with growling and snapping. A recent bite to the caregiver prompted referral. Red flags were present in resting, self-grooming, locomotion, solo and social play, and caregiver interactions. The caregiver questionnaire and home videos confirmed these concerns. Mapping the timeline showed normal socioemotional functioning in the first 2 years, followed by a gradual onset of behavioral signs, suggesting a progressive issue rather than an emotional or cognitive disorder. The pattern was suggestive of an underlying painful process. A CT scan revealed osteochondrosis dissecans of L7 with cauda equina compression and right-sided foraminal stenosis. Treatment included an orthopedic bed for physical comfort, gabapentin (20 mg/kg TID) for maladaptive pain, and fluoxetine (1.5 mg/kg SID) for socioemotional impairment and compulsive licking. After 6 months, red flags decreased, but 3 years later, the dog developed acute lymphoma and was euthanized at age 10. Challenges included the dog’s reluctance to walk during observation and growling/snapping during the clinical exam. Licking and biting were initially interpreted as purely behavioral, rather than indicative of an underlying painful condition, and the absence of radiographic abnormalities reinforced the assumption that no pain was present.

### Dog 2: Sheltie, M, 8y

Dog 2 was referred for persistent vocalizing at night and sleeping problems over the past 4 months, including panting, vocalizing, and scratching at doors. Medical history, GP clinical exam, neurological evaluation, direct observation, and spinal radiographs revealed no abnormalities. The referral was based solely on the vocalizing and sleeplessness. Red flags were identified in resting behavior, sleeping behavior, and caregiver interactions, specifically vocalizing when the caregivers went to sleep. Timeline mapping showed normal sleep and rest patterns for 7.5 years, with a sudden change 4 months earlier. The abrupt onset did not suggest an emotional or cognitive disorder but was highly indicative of an unidentified painful process. A CT scan revealed cervical disc bulging at C3-C4 with ventral spinal cord compression, degenerative joint disease of the left hip, and lumbar disc bulging at T12-T13 with ventral spinal cord compression. Treatment focused on physical comfort with an orthopedic bed and maladaptive pain management with gabapentin (20 mg/kg TID). To address impaired socioemotional functioning and disrupted sleep, sertraline (2 mg/kg BID) was prescribed. After 6 months, the dog showed recovery, and 6 years later, no red flags remained. Dog 2 continues to sleep well at age 17 while remaining on medication. Challenges included normal gait during observation, a reluctance to be handled, and a tense but pain-free presentation during the clinical exam. The GP initially interpreted nighttime vocalizing as a “learned behavior,” delaying recognition of the underlying painful condition, and the absence of radiographic abnormalities reinforced the assumption that no pain was present.

### Dog 3: Golden Retriever, FS, 3y

**Figure 2:**
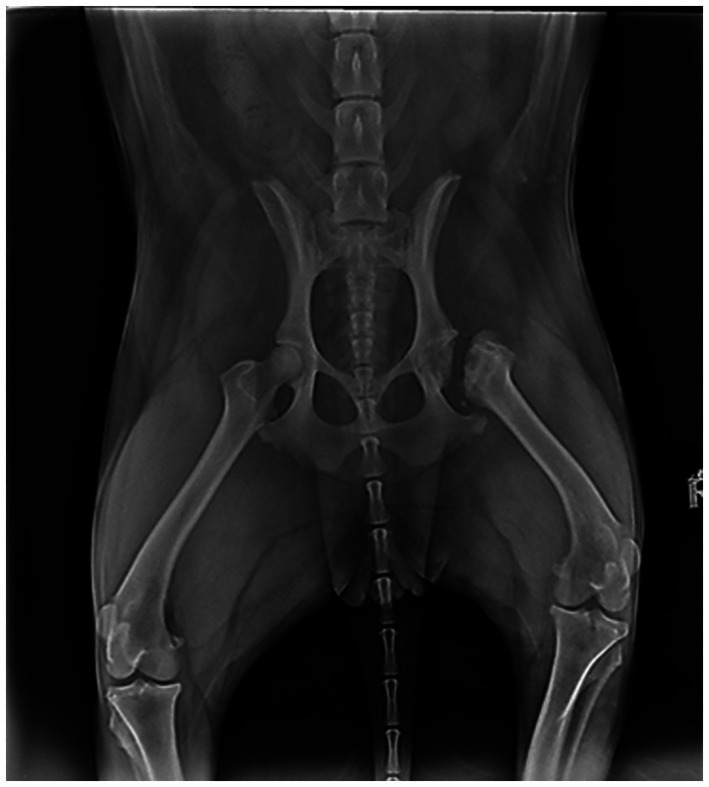
Dog 3: Post operative radiograph showing the right sided femoral head resection.

**Figure 3:**
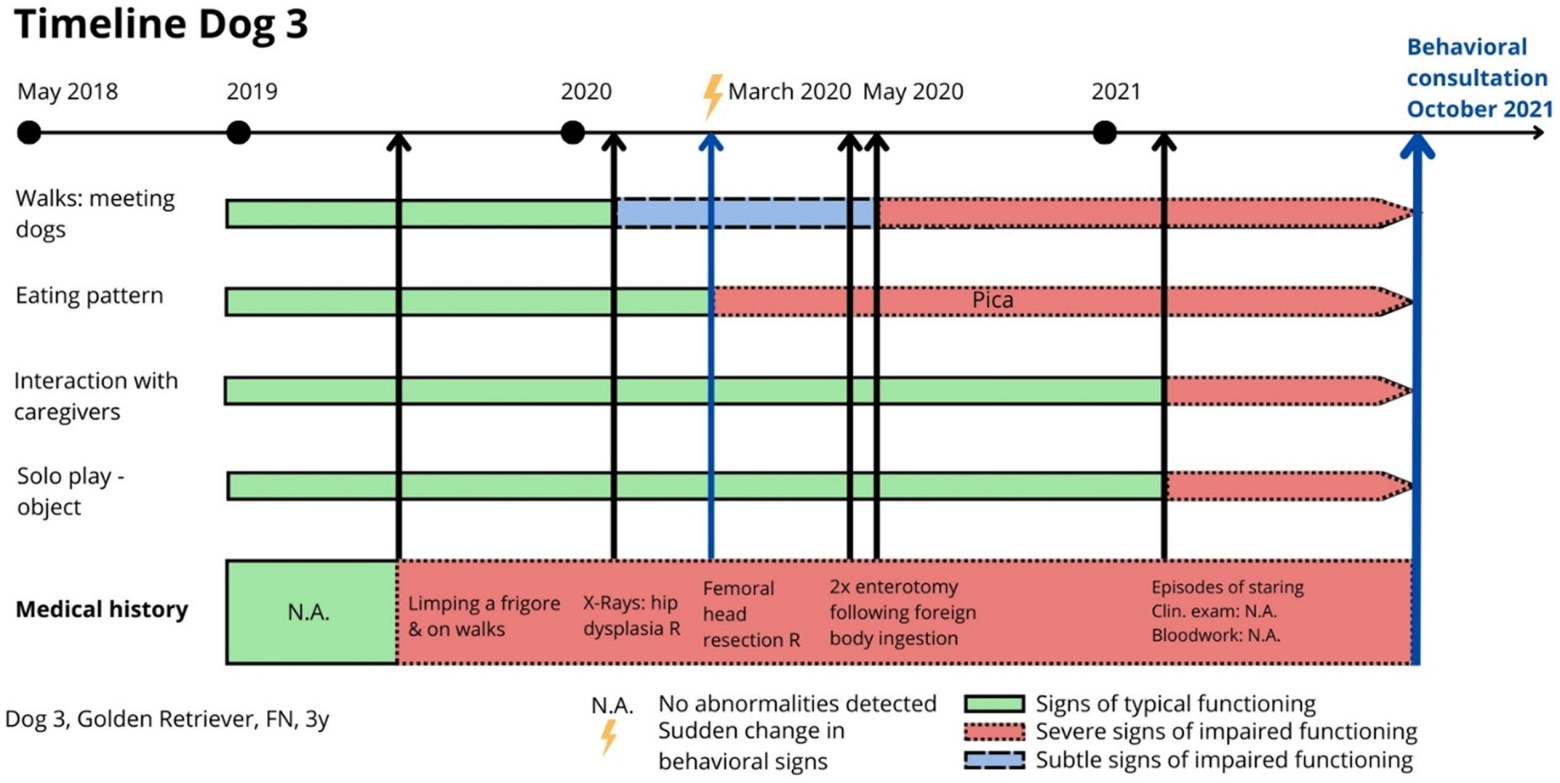
Dog 3: Timeline indicating relevant clinical signs (red flags) relating to physical and emotional health, helping the clinician to differentiate and suspect the existence of painful processes.

Dog 3 had a history of painful right hindlimb lameness 2 years prior to the behavioral exam. Radiographs revealed hip dysplasia in the right hindlimb, and femoral head osteotomy was performed. Post-surgery, direct observation and radiographs showed no abnormalities (Figure 2). Shortly after the hip surgery, the dog underwent two enterectomies following the ingestion of foreign objects. The dog presented red flags in multiple behavioral contexts, including eating (pica), resting (standing and staring instead of lying down), locomotion (reluctance to walk), play (absence of play), interaction with caregivers (isolation), and walks (lunging at dogs). When mapping the chronology of these red flags onto a timeline (Figure 3), it became clear that social behavior toward other dogs had been typical for the first 1.5 years of life but began deteriorating around the onset of lameness. Pica behavior developed immediately after the femoral head osteotomy, while changes in interaction with caregivers and play only emerged more recently. These findings strongly suggested a link to maladaptive pain rather than an emotional or cognitive disorder. Treatment focused on optimizing physical comfort with an orthopedic bed, physiotherapy, and weekly swimming. Pregabalin (2 mg/kg BID) was prescribed for maladaptive pain, and fluoxetine (1.5 mg/kg SID) for impaired socioemotional functioning. At 6 months, all red flags except avoidance of other dogs had resolved. However, 4 years later, the dog developed Progressive Retinal Atrophy (PRA) and was euthanized at age seven due to an abrupt recurrence of emotional distress linked to blindness. Challenges were that during observation, the dog walked normally with no visible lameness. The clinical examination revealed reluctance to be handled, a tense body, and no overt signs of pain. The behaviors related to pica, lunging at dogs, decreased play, and isolation were initially misinterpreted as behavioral issues. Because the surgery and healing process proceeded normally, post-operative pain was not initially considered.

### Dog 4: Labrador, M, 8y

Dog 4 had a history of intermittent limping for several years. The GP clinical exam revealed that the dog would growl and snap when the hind end was touched. Direct observation showed signs consistent with hip dysplasia, and radiographs confirmed bilateral hip dysplasia. The dog was referred for behavioral assessment due to vocalizing at night and sleeping problems. Several red flags were identified, including altered resting behavior (restlessness, frequent position changes day and night), sleep disturbance (vocalizing), increased self-grooming (focused on the hind end), locomotion difficulties (trouble getting up, particularly in colder temperatures), loss of play behavior (both object play and social play with caregivers and the family dog), withdrawal from social interactions, and avoidance during walks (keeping distance). Mapping the chronology of these flags revealed that the decline in socioemotional functioning had only occurred in the last 6 months. These findings did not indicate an emotional or cognitive disorder but strongly suggested maladaptive pain. Treatment focused on physical comfort with an orthopedic bed, gabapentin (20 mg/kg TID) for maladaptive pain, and fluoxetine (1.5 mg/kg SID) for impaired socioemotional functioning. After 6 months, red flags were reduced, play and interaction resumed, and sleeping patterns improved. Four years later, the dog was euthanized at age 12 due to unrelated medical complications. Direct observation confirmed a history of hip dysplasia, and limping was considered expected. During clinical examination, the dog was reluctant to be handled, lying down and growling when touched on the hind end. The insomnia, restlessness, vocalizing, and altered social interactions were initially misinterpreted as behavioral issues.

### Dog 5: Bulgarian Scenthound, M, 7y

Dog 5 had undergone right hindleg amputation due to a malignant peripheral nerve sheath tumor (MNST) 6 months prior. The GP clinical exam showed that the surgical site had healed as expected, with no abnormalities. The dog was referred for behavioral concerns, including vocalizing at night and sleeping problems. The dog displayed red flags across multiple contexts: eating (reluctance to touch food), elimination (urination in the home), resting (restlessness, vocalizing), sleeping (disturbed, vocalizing), and self-grooming (increased licking at the surgery site). Play and interaction with caregivers and the family dog were minimal or absent. Locomotion was functional but slow due to the three-legged gait. Mapping the chronology of these signs onto a timeline revealed a gradual decline in day-to-day functioning a few weeks after amputation, with no signs of recovery. These findings strongly suggested post-surgical maladaptive pain rather than an emotional or cognitive disorder. The dog was given an orthopedic bed for physical comfort and was treated with gabapentin (20 mg/kg TID) for maladaptive pain, and fluoxetine (1.5 mg/kg SID) for impaired socioemotional functioning. After 6 months, all red flags were reduced, with improved sleep and resumed play and interaction. However, at 2 years, the MNST recurred, and the dog was euthanized at age nine. Challenges were that during direct observation, increased licking at the healed surgical site was initially seen as normal post-operative behavior. The clinical examination revealed no skin damage or signs of pain. Vocalizing, social isolation, irritability, and urination were initially misinterpreted as purely behavioral.

### Dog 6: Corgi, M, 2y

**Figure 4:**
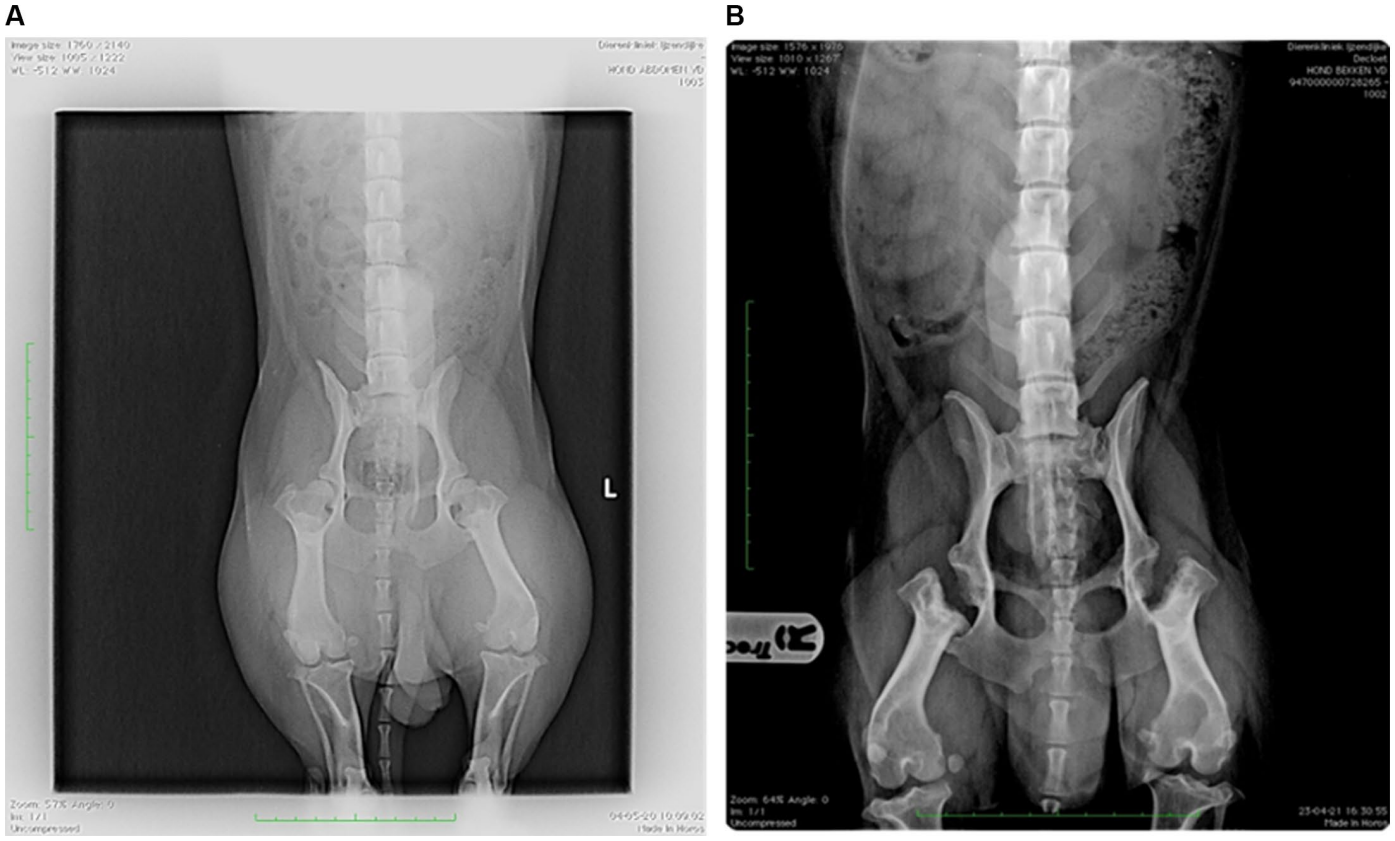
Dog 6: **[A (left)]** Preoperative radiograph showing bilateral hip dysplasia and **(B)** postoperative radiograph showing bilateral femoral head resection.

Dog 6 had a history of hind end limping since the age of one. Radiographs revealed bilateral hip dysplasia (Figure 4A), leading to femoral head resection on the right hind limb, followed by the left 4 months later. Post-surgery radiographs showed no abnormalities (Figure 4B), and revalidation appeared normal. However, weeks after the second surgery, Dog 6 began attacking the family dog and was referred for “aggression.” Dog 6 displayed red flags across multiple contexts: decreased eating, increased resting (lying or sitting all day), disturbed sleep (restlessness), difficult locomotion (whining), ceased play, interaction with the family dog became defensive (attacking on sight and injuring), interaction with caregivers diminished (isolation), and walking became impossible. Mapping the chronology of these signs onto a timeline revealed that the dog’s decline began 1 week after the second surgery, with no prior history of similar issues. The absence of recovery strongly suggested maladaptive pain rather than an emotional or cognitive disorder. Treatment included strict separation from the other dog, an orthopedic bed for comfort, gabapentin (20 mg/kg TID) for maladaptive pain, and fluoxetine (1.5 mg/kg SID) for impaired socioemotional functioning. However, no improvement was observed, and treatment was discontinued by the caregiver. The dog was ultimately euthanized. During direct observation, limping post-surgery was seen as part of normal revalidation. Because the surgery and healing process proceeded normally, post-operative pain was not initially considered. Clinical and radiological exams showed no abnormalities. Increased irritability, withdrawal from caregivers, and apathy were initially misinterpreted as purely behavioral.

### Dog 7: Labrador mix, M, 9 months

**Figure 5:**
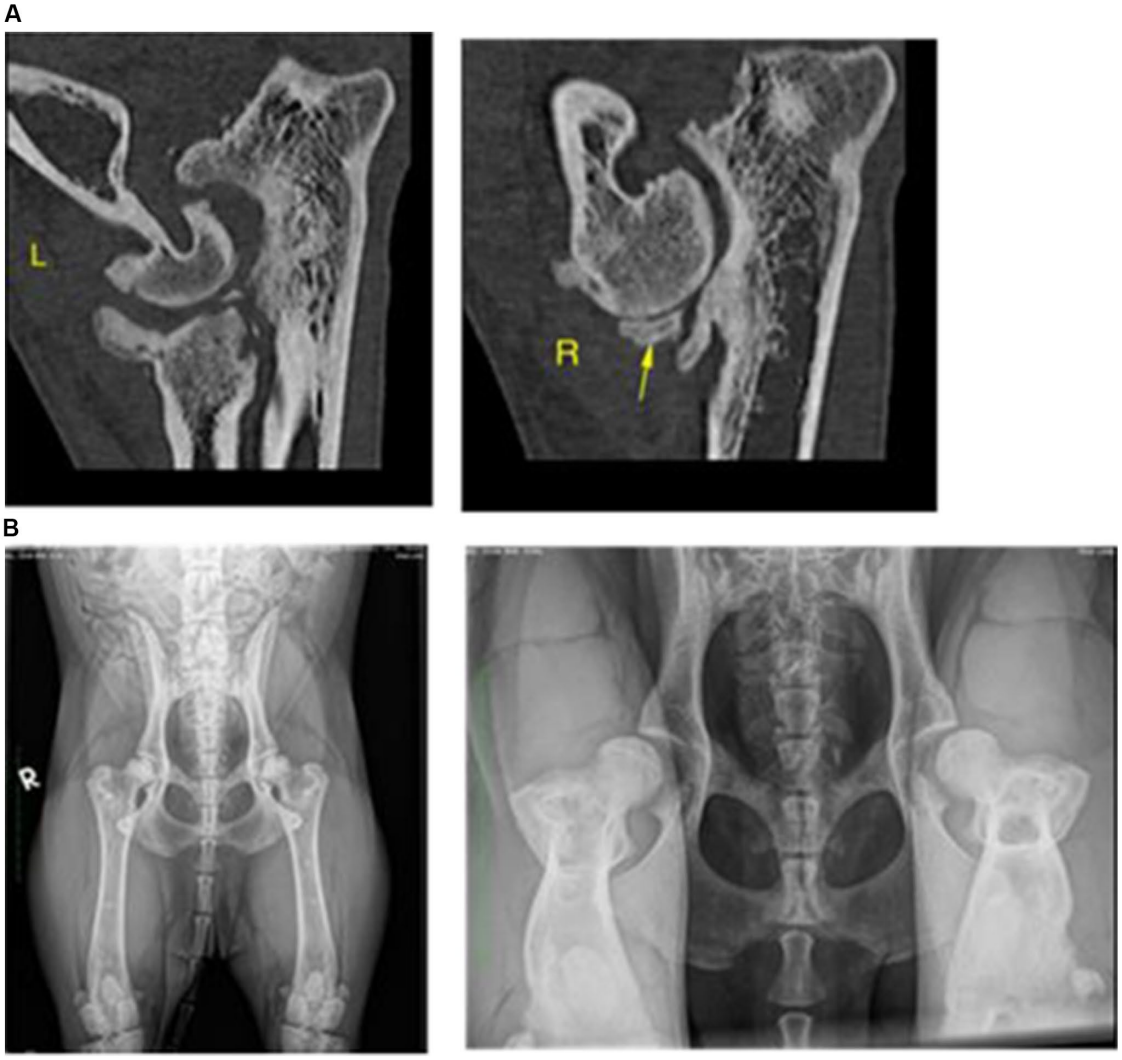
Dog 7: **[A (above)]** CT-scan showing bilateral elbow dysplasia and **(B)** Radiographs showing bilateral hip dysplasia.

Dog 7 had a history of five enterectomies due to foreign body ingestion and intermittent limping since puppyhood. The GP clinical exam revealed painful front and hindlimbs, and direct observation confirmed limping on all four limbs. An orthopedic exam at 7 months indicated painful elbows and hips, with CT-images and radiographs revealing bilateral elbow dysplasia and severe bilateral hip dysplasia (Figure 5). The dog had been on NSAID treatment and was referred for pica. Dog 7 displayed red flags across multiple contexts: eating behavior (pica), resting behavior (lying down most of the time), and locomotion (limping on all four limbs). Pica was specifically observed when caregivers or the family dog were absent. Mapping the chronology of these red flags onto a timeline showed no prior signs of emotional dysfunction, suggesting a strong correlation between pica and maladaptive pain. Given the caregivers’ reluctance to pursue surgery, treatment focused on improving physical comfort with an orthopedic bed and managing maladaptive pain with gabapentin (20 mg/kg TID) as an add-on to the NSAID treatment. At 6 months, pica had resolved. At 2 years, the dog remained on gabapentin. Challenges during direct observation were that the dog’s persistent limping aligned with the medical findings. During clinical examination, painful elbows and hips were found, which was consistent with radiographic imaging. However, pica was not initially linked to pain because the dog already received NSAIDs and pica was instead interpreted as purely behavioral.

### Dog 8: Border Collie, M, 3y

**Figure 6:**
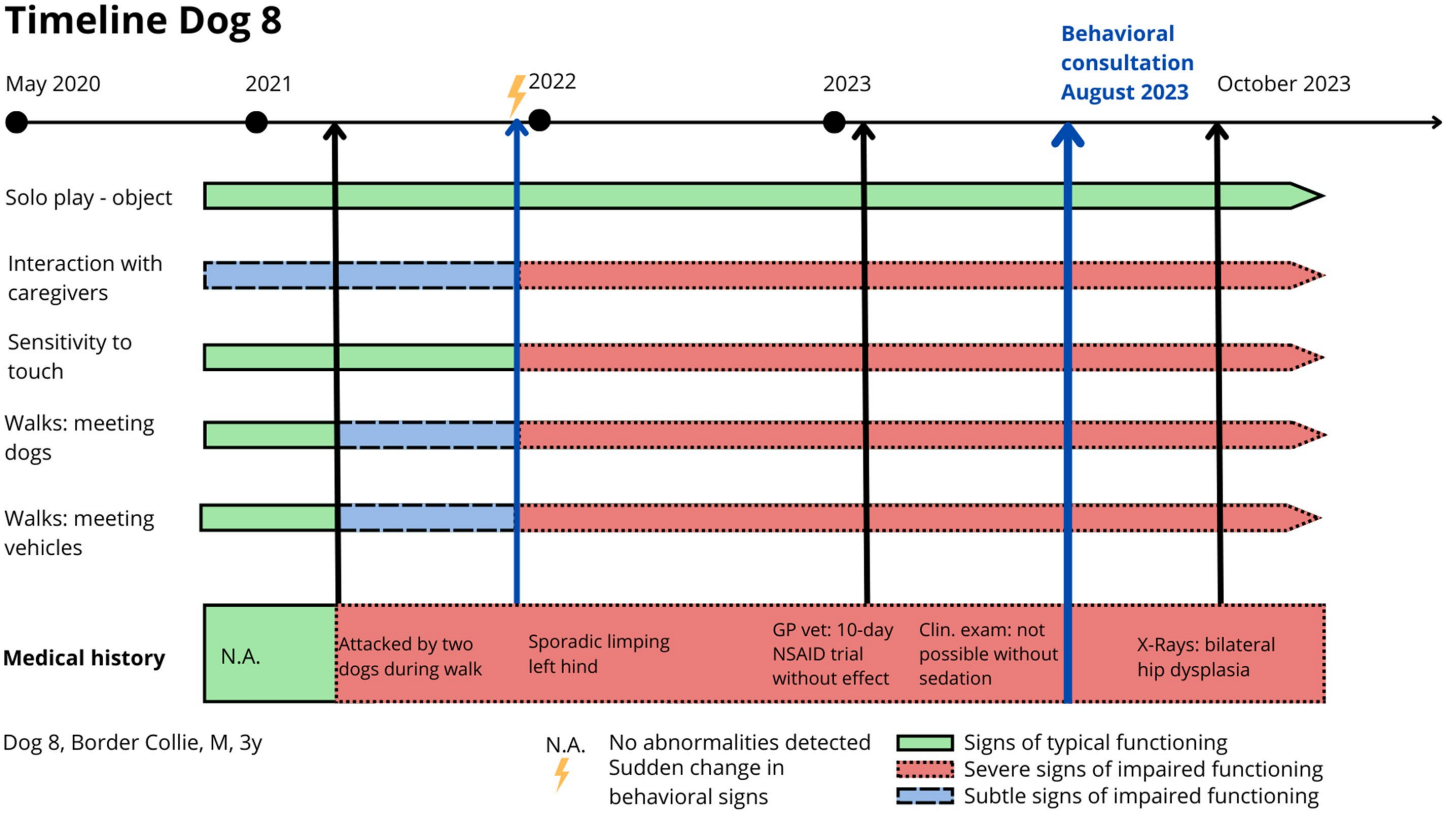
Dog 8: Timeline indicating relevant clinical signs (red flags) relating to physical and emotional health, helping the clinician to differentiate and suspect the existence of painful processes.

**Figure 7:**
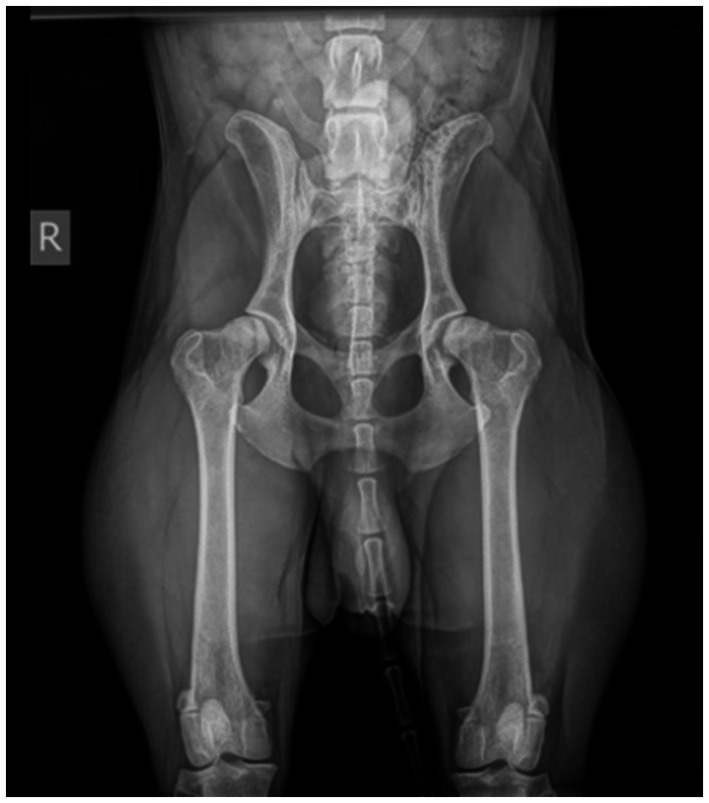
Dog 8: Radiograph showing bilateral hip dysplasia with remodeling of the left femoral head and neck.

Dog 8 had a history of intermittent left hindlimb limping, which was not deemed relevant due to the dog’s otherwise active appearance. The GP clinical exam revealed that the dog would growl and snap when touched. The dog was referred for biting family members. Dog 8 displayed red flags in multiple contexts: resting behavior (lying near caregivers but biting when touched), sleeping behavior (growling when approached), locomotion (intermittent limping), play with caregivers (reduced), interaction with caregivers (defensive when approached or touched), and walking (lunging at dogs and vehicles). Mapping the chronology of these red flags onto a timeline (Figure 6) showed a gradual regression in social behavior, worsening from subtle avoidance to severe defensive reactions. The abrupt onset of touch sensitivity strongly suggested a red flag for maladaptive pain, warranting further diagnostics. Orthopedic evaluation under sedation showed no abnormal range of motion in the hip joints, but radiographs (Figure 7) revealed significant bilateral hip dysplasia with remodeling of the left femoral head and neck. The caregivers declined surgery, physiotherapy, or additional affective health support. Treatment focused on raising awareness of behavioral signs as red flags for pain and optimizing comfort. Management included a private resting area and avoiding other dogs. Pain control consisted of robenacoxib (4 mg/kg SID for 2 months) and gabapentin (20 mg/kg TID). Challenges were that direct observation revealed a normal gait with no limping during consultation. Clinical examination was challenging without sedation, as the dog growled, snapped, and resisted muzzling. The GP initially misinterpreted biting as a behavioral issue after a failed 10-day NSAID trial. The dog’s young age (3 years) and active lifestyle reinforced the assumption that pain was not a factor.

### Dog 9: Malinois, M, 8y

Dog 9 was referred for panic attacks triggered by noises and recent sleeping disturbances. The medical history showed no abnormalities. The GP clinical exam and direct observation were unremarkable, and the orthopedic exam revealed no findings. Radiographs of the hip and back regions were also normal. Dog 9 displayed red flags in multiple contexts: resting behavior (panting, trembling), sleeping behavior (restlessness, vocalizing), and interaction with caregivers (distancing and escape attempts when hearing noises, both loud and silent). Recently, the dog began panicking both at home and on walks, even in the absence of noises. Mapping the chronology of red flags onto a timeline showed a long-standing noise sensitivity (trembling, hiding) that had abruptly worsened over the past 2 months, escalating into panic attacks, climbing on furniture, and escape attempts. Given this progression, the sudden escalation strongly suggested a red flag for maladaptive pain, warranting further diagnostics. Orthopedic examination revealed no abnormalities, but a CT scan identified high-grade degenerative lesions, calcifications, and disc bulging with spinal cord compression at multiple levels (cervical, thoracic, and lumbar). Additionally, impingement was noted at the attachment of the right biceps tendon. Before this diagnosis, several medications were trialed to reduce panic attacks in this dog. Initial treatment with fluoxetine (2 mg/kg SID) and gabapentin (20 mg/kg TID) showed no response. The addition of trazodone (SARI) did not lead to improvement. Following the CT scan results, treatment was adjusted to amitriptyline (TCA) and pregabalin (4 mg/kg BID), which resulted in significant improvement. Panic attacks ceased both during the day and at night, though the dog remained sensitive to loud noises, displaying trembling. Challenges were that direct observation revealed a normal gait during consultation, and clinical examination showed no overt signs of pain. The increasing noise sensitivity, sleeplessness, panting, and panic reactions were initially misinterpreted as purely behavioral. However, further investigation revealed underlying physical conditions, including spinal cord compression and shoulder tendon impingement.

### Dog 10: Husky, M, 8y

Dog 10 had a history of separation-related problems, intolerance of restraint, and noise sensitivity since adoption at 1.5 years old. Intermittent left front limb limping had been noted, but clinical, orthopedic, and radiographic examinations showed no abnormalities. Despite persistent limping, steroid infiltrations, and physiotherapy were the only interventions until the dog was referred for recently snapping at the caregiver during walks. Over 5 years, behavioral issues related to noise and social interactions remained stable until they worsened abruptly a year before the behavioral examination. Dog 10 displayed red flags across multiple contexts: eating behavior (reduced appetite), resting behavior (restless, panting), sleeping behavior (waking up at night and vocalizing), self-grooming behavior (excessive licking of the front paw), locomotion (limping after rest), play behavior (loss of solo- and social play), interaction with caregivers (episodes of social isolation and episodes of seeking attention), and behavior during walks (snapping towards caregiver when meeting dogs, people, or vehicles).

**Figure 8:**
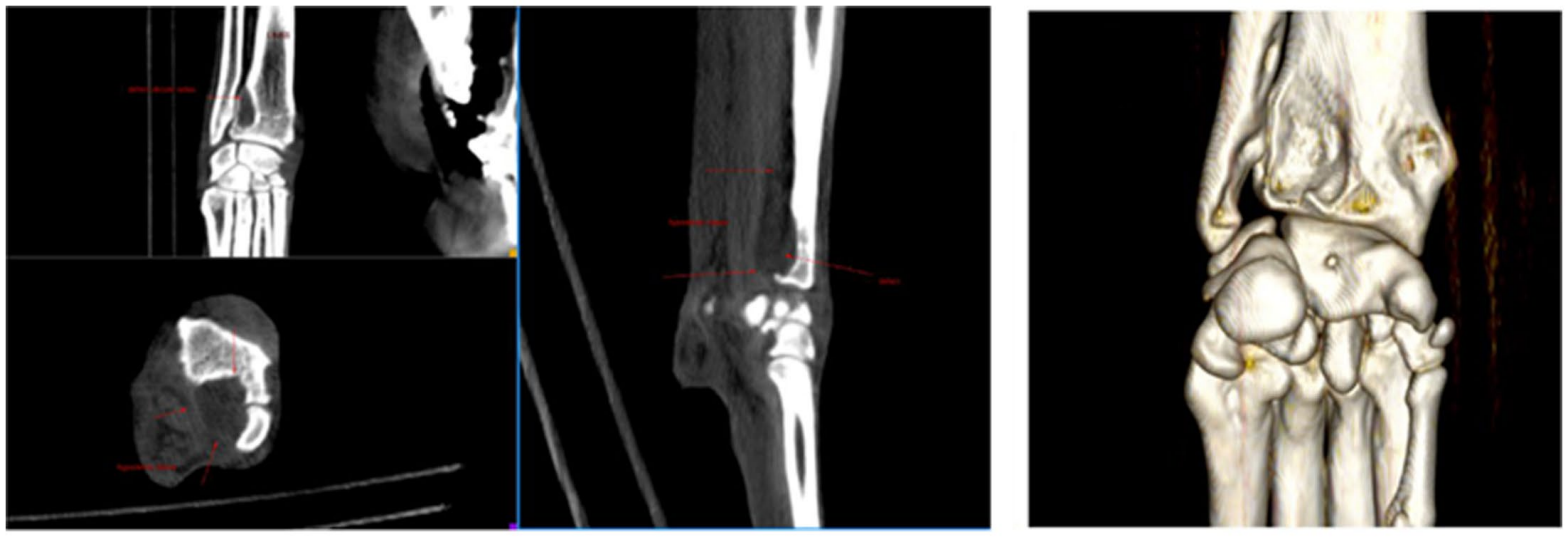
Dog 10: CT images of the left front paw displaying lesions suggestive of a malignant peripheral nerve sheath tumor (MNST).

Given the chronic but stable behavioral impairments and their recent escalation, pain was suspected as a contributing factor. A CT-scan revealed the suspicion of a malignant peripheral nerve sheath tumor in the left front leg (Figure 8). With worsening welfare and escalating pain, euthanasia was chosen as the most humane option. Challenges were that the dog had presented a tense posture, normal gait, and resistance to handling during consultation, which led to the initial interpretation of signs as purely behavioral.

## Discussion

Diagnosing maladaptive pain in dogs referred for behavioral complaints presents multifaceted challenges, particularly when pain primarily manifests as behavioral signs (Frank, 2014; Mills et al., 2020, 2023). While the link between maladaptive pain and behavioral signs is well-established and has been described in previous literature, distinguishing between purely emotional signs and behavioral signs of maladaptive pain, whether in terms of comorbidity or causality, remains a significant challenge (Mathews et al., 2014; Mills et al., 2020).

The aim of this study was to describe specific challenges encountered when diagnosing dogs presenting with behavioral complaints and to provide practical screening tools to assess whether clinical signs are indicative of a behavioral disorder, a maladaptive pain process, or a combination of both.

The Toolbox approach integrates caregiver questionnaires, home videos, and a timeline into the behavioral examination to help detect possible red flags that might be indicative for the presence of maladaptive pain (Fatjó and Bowen, 2020; De Keuster, 2025, unpublished).

The descriptive behavioral diagnosis in the sample of 10 dogs consisted of ‘impaired socioemotional functioning’ toward social or nonsocial stimuli (Fatjó and Bowen, 2020). Interestingly, behavioral signs in Group 1 could not be explained by behavioral history and could not be explained by an underlying emotional disorder or cognitive dysfunction. In these seven dogs, behavioral signs were highly indicative of unidentified maladaptive pain (Table 4). In Group 2, behavioral signs were only partially linked to their history of impaired socioemotional functioning. In these three dogs, an acute worsening of behavioral signs at a moment in time was found highly indicative for unidentified maladaptive pain (Table 5). Based on these findings, the authors identified three potential areas of bias in the screening process of dogs presenting with behavioral complaints: bias in the patient observation, bias in clinical examination, and bias in the interpretation of clinical signs, as summarized in Table 3.

## Challenges

### Observational bias

Observation plays a crucial role in diagnosing pain in dogs with behavioral complaints, yet it presents several potential biases in the case of detecting maladaptive pain. The first factor relates to the context in which the patient’s behavior is observed. As the expression of a dog’s behavior is context dependent, behavioral, and physical signs may significantly differ between a home setting and a clinical environment (Monteiro et al., 2023). In addition, factors like perceived stress can either amplify or diminish the subtle nature of clinical signs, thereby limiting the accuracy of in-clinic observations (Monteiro et al., 2023). In our sample (*n* = 10), dogs 2, 3, 5, 8, 9, and 10 represented this type of in-clinic observational bias, since each of the dogs displayed normal gait during observation in the consultation room, despite exhibiting (sporadic) limping or stiffness in the home setting. Conversely, in some dogs, direct observation may be hampered because a dog refuses to walk, such as dog 1: he lied down and refused to get up, hereby hindering gait analysis and representing a major challenge for the clinician.

To overcome observational in-clinic bias, research suggests that caregiver-reported observations should be seen as a crucial cornerstone in the diagnostic process (Wiseman-Orr et al., 2006; Reid et al., 2018; Malkani et al., 2024). As noted by Reid et al. (2018), caregivers are known to be the best observers of their dogs’ altered functioning, given their familiarity with the animal’s typical patterns and changes. Further, Malkani et al. (2024) points out that while owners easily identify physical signs of pain, they may overlook subtle behavioral changes, even though these often precede physical symptoms (Reid et al., 2018; Malkani et al., 2022, 2024). These conclusions are supported by the AAHA 2015 guidelines for pain recognition, indicating that caregivers easily may recognize signs of acute pain, but often fail to identify chronic pain. In our case sample (*n* = 10), direct observation also represented a challenge for referring veterinarians, since it did not lead to a suspicion of a painful process as a cause of behavioral signs in any of the dogs (Epstein et al., 2015).

### Clinical examination bias

Detecting maladaptive pain in dogs during clinical examination presents inherent difficulties, particularly because signs of maladaptive pain may not always be present or detectable at the time of examination (Mathews et al., 2014; Reid et al., 2018; Monteiro et al., 2023; Malkani et al., 2024). Additionally, defensive responses, such as growling, snapping, or biting, originating from an underlying emotional disorder or the painful process itself, may hinder or prevent a thorough clinical examination.

In our sample (*n* = 10), clinical examination by the referring veterinarian was reported to be particularly difficult for five dogs. Dogs 4, 6, 8 and 10 all displayed biting when touched, and were perceived as risky for the team. On the other hand, the clinical examinations of dogs 2 and 3 showed no abnormalities, while the examination of dogs 5 and 9 was challenging due to continuous trembling, restlessness, and whining. Dog 7 was the only dog in our sample to display a typical (acute) pain response (whining, screaming) when the elbows and hips were palpated.

### Interpretation bias of clinical signs

One of the biggest challenges in diagnosing maladaptive pain in dogs presented with behavioral complaints is distinguishing between emotional and physical causes of the behavioral signs. The current literature provides limited guidance on differentiating behavioral signs resulting from maladaptive pain from those related to purely emotional problems, leaving a significant gap in clinical practice. Misinterpretations often arise when clinical signs lack clear manifestations or diagnostic evidence, such as abnormal imaging findings or identifiable lesions. In such cases, veterinarians may prematurely attribute behavioral signs to emotional causes. Recent research highlights that stress and nociception interact in complex ways, with anesthetic and surgical stress influencing behavioral and physiological responses in dogs. These findings underscore the need for a multimodal approach to pain assessment (Hernández-Avalos et al., 2021).

In our caseload, for example, dogs 1 and 2 exhibited behavioral changes (respectively lick granuloma and sleeping problems), but without apparent signs of limping. Because initial radiographs revealed no abnormalities, pain was excluded as a differential diagnosis and both dogs were referred for “behavioral complaints.” Interestingly, the outcome of the behavioral assessment revealed no indications of underlying emotional disorder, and from the behavioral perspective, clinical signs in both dogs were seen as highly indicative of unidentified maladaptive pain. In this way, the authors prompted further investigation, and in both dogs, CT results uncovered specific lesions that were found to support the hypothesis of maladaptive pain. The next example is dog 9. This dog displayed severe panic reactions to noises without obvious physical signs of gait abnormality. As the panic attacks deteriorated over time, without any response to behavioral treatment, the dog was referred to medical imaging, where a CT scan revealed significant back lesions consistent with maladaptive pain. Notably, research has already indicated a link between signs of noise sensitivity or panic attacks, and underlying maladaptive pain (Fagundes et al., 2018; Amadei and Pierantoni, 2022). A more complex example is dog 10, which had a history of longstanding behavioral problems and suddenly exhibited a deterioration of behavioral signs. The dog showed no signs of limping but was difficult to examine due to defensive responses. While radiographs of the dog’s left thoracic limb showed no abnormalities, the behavioral assessment suggested maladaptive pain. Referral to a radiologist and subsequent CT imaging revealed a lesion consistent with a malignant nerve sheath tumor.

These cases underscore the risk of excluding pain as a cause for behavioral signs in dogs without overt physical symptoms like limping or detectable radiographic abnormalities. Clinical signs of osteoarthritis, for instance, often fail to correlate with the severity of radiographic findings (Hielm-Björkman et al., 2003; Enomoto et al., 2024). Relying solely on clinical examinations and imaging to rule out pain may result in missed diagnoses and inadequate treatment for pain-driven behavioral changes. While the absence of abnormalities on imaging does not rule out pain, comprehensive diagnostic imaging (radiographs, MRI, CT scans) plays a vital role in identifying underlying conditions contributing to maladaptive pain, such as intervertebral disc disease (da Costa et al., 2020). Imaging was employed in 8 of the 10 cases, revealing various causes of chronic pain, shown in Tables 4, 5.

Certain behavioral signs, such as pica, are often misinterpreted or dismissed as ‘purely behavioral.’ For example, dogs 3 and 7, both of which underwent multiple enterectomies, highlight that pica can also be associated with musculoskeletal pain rather than solely gastrointestinal or behavioral issues (Mills et al., 2020).

Interpretation bias is also common with signs such as sleep disturbances or behavioral changes in senior dogs. Signs like waking up at night or vocalizing when separated from caregivers may be incorrectly attributed to cognitive dysfunction or “just old age,” while maladaptive pain should always be considered as a differential diagnosis (Mondino et al., 2021; Monteiro et al., 2023). In our sample, dogs 2, 4, and 5 displayed vocalizing at night and sleep disturbances. Behavioral evaluations revealed these signs were not attributable to an emotional or cognitive disorder but were highly indicative of undiagnosed maladaptive pain.

Short-term NSAID trials to confirm pain can also be misleading. In dog 8, a 10-day NSAID course failed to alleviate symptoms, leading to the incorrect conclusion that pain was absent. This example stresses the need for a multimodal approach in cases of suspected maladaptive pain, incorporating multiple analgesics and adjunct therapies (Epstein et al., 2015; Monteiro et al., 2023). A negative response to a single pain trial should prompt further diagnostics rather than excluding pain.

## Opportunities: practical tools used in behavioral examinations

A Toolbox approach combines multiple sources of information, integrating medical history, caregiver questionnaires, home videos, and caregiver interviews to map relevant clinical signs in chronological order. This approach can aid in identifying maladaptive behavioral functioning (red flags), helping clinicians differentiate and diagnose underlying physical, emotional, or cognitive dysfunction.

Caregiver questionnaires consist of open-ended questions, providing the opportunity for caregivers to describe what they observe in their dog, rather than interpreting what is happening. The behavioral questionnaire involves questions about a dog’s day-to-day behavior in the home setting and on walks. The integration of open-ended questions allows for capturing nuanced caregiver information about the history and day-to-day functioning of the dog.

While clinical examination is considered a valuable diagnostic tool, our dog sample demonstrates that results from clinical examination should not be seen as ‘key’ in pain patients, but rather as part of a more comprehensive diagnostic approach. An important consideration is that a normal or limited clinical examination should not automatically lead to the conclusion that pain is absent. Instead, clinical findings should be integrated within a broader diagnostic framework.

To address the challenges of context-dependent observations, the authors suggest implementing home video assessment, to evaluate a dog’s day-to-day functioning in a familiar setting. In the authors’ approach, caregivers are asked to record common contexts, such as eating, drinking, resting, eliminating, self-grooming or sleeping behaviors, as well as social interactions with family members or dogs, or behaviors on walks toward people, pets, and vehicles. These recordings often reveal subtle physical and behavioral signs, such as gait abnormalities, posture changes, or stiffness, which might highlight pain-related or maladaptive behaviors. To the authors’ experience, the above behaviors may be overlooked when solely relying on the caregivers’ description, or on in-clinic evaluations. This approach aligns with WSAVA guidelines (2023), and the AAHA guidelines (2022) that recommend using home video recordings to maximize success in pain detection (Gruen et al., 2022; Monteiro et al., 2023).

By identifying and monitoring green and red flags as indicators on a timeline, insights into the potential links between observed behaviors and underlying pain can be easily gained. Timelines are essential for tracking the onset and progression of signs, allowing for identification of patterns suggestive of maladaptive pain (as illustrated for dogs 3 and 8, respectively, in Figures 1, 3). In cases where behavioral and physical signs can be linked on a timeline, in the absence of a behavioral history, the connection strongly suggests that behavioral signs may be related to pain. In cases where a sudden escalation of behavioral signs occurs on the timeline of a dog with a known behavioral history, the escalation should be seen as a reason for further investigation and screening for painful processes.

In order to avoid prematurely attributing behavioral complaints solely to emotional problems, the interpretation of clinical signs should occur after comprehensive evaluation of behavioral and medical history alongside questionnaires, and video observation. The authors stress that behavioral signs always need to be interpreted within the framework of a timeline. Following this approach, a caregiver interview is conducted to verify and cross-check the interpretation of observed signs. Based on the combined information, a list of potential differential diagnoses is made, considering both physical and behavioral causes. In our sample (*n* = 10), all differentials of suspecting an underlying painful process were made thanks to this approach.

Awareness among veterinary professionals about the potential pitfalls of behavioral signs relating to maladaptive pain should be seen as essential for early detection and effective management. Educating professionals to recognize these signs and encouraging veterinary teams to include maladaptive pain in their differential diagnoses may facilitate earlier interventions and better outcomes, enhancing the welfare and quality of life for affected dogs.

## Limitations and future recommendations

Limitations of this study include a small sample size and potential sample selection bias, as participants were not randomly selected. The lack of previous research on this topic and the absence of validated tools for assessing behavioral disorders and chronic pain in such patients further constrained the study. Consequently, the approach relied on clinical reasoning. These limitations, including the lack of statistical analysis and standardized methods, may limit the generalizability of the findings. Future studies should aim to include larger, more diverse samples and employ statistical analysis to enhance the reliability of results. Another limitation of this study is that the study design did not include the recognition and evaluation of facial action units (FAU’s). FAU-based analysis has been increasingly recognized as a valuable tool for assessing emotional states and pain in animals, offering a more objective and standardized approach to pain evaluation. Incorporating this methodology in dogs may improve the identification of subtle pain-related facial expressions, thereby enhancing diagnostic accuracy and clinical decision-making (Mota-Rojas et al., 2021).

## Conclusion

Diagnosing maladaptive pain in dogs presenting with behavioral problems requires a comprehensive approach. The use of a detailed questionnaire, home videos, and a timeline allows for a more thorough assessment of the dog’s behavior, while advanced diagnostic imaging may uncover occult pain that may not be visible during routine examinations. By recognizing red flags of maladaptive pain, and raising awareness about the subject, clinicians can intervene earlier and implement multimodal treatment strategies that address both behavioral and pain related symptoms. Ultimately, the integration of this diagnostic approach can improve the quality of life for affected dogs and their caregivers, reducing the duration of suffering and facilitating more effective management of complex behavioral cases.

## Data Availability

The original contributions presented in the study are included in the article/supplementary material, further inquiries can be directed to the corresponding author.
